# The bitten man: Reflections on international health

**Published:** 2007-10-22

**Authors:** Simon Oczkowski

## Away

Tucked away somewhere in the twisting innards of Mulago Hospital, Kampala, Uganda, there lies the pink, neatly stapled medical file of a man who is doomed to die. I know this because I saw him on Thursday night.

“There’s an interesting case you should see if you have time,” said the attending physician cheerily. “You should look up his condition in your book and take a history. It might be good for a case write-up when you get home.”

We had just stepped into the casualty department, hoping to catch some of the evening’s action. It seemed as though the action had found us. Puzzled, one of us asked what the patient had.

“Rabies, a classic case,” the physician said. She paused. “But I’m not sure what to do about it.” Having had her say, she closed the door to the treatment room behind her, leaving us alone in the crowded hallway.

We had been in Kampala for only two weeks but already we had seen our fair share of “interesting cases” — that is to say, we had seen dozens of people whose disease had progressed to the point where their clinical findings were evident even in our clumsy hands: young diabetics with histories of multiple ketoacidosis because they could not afford insulin, children with spleens swollen from chronic malaria, and wasted people of all ages exhibiting the innumerable and tragic complications of HIV infection. At home in Canada, patients would rarely, if ever, present at such advanced stages of disease. Here, they were the rule.

To see these patients — cases — day after day was, of course, challenging. The initial shock of being thrust into a seemingly chaotic environment where the standards of care were so dramatically different from what we were used to was almost overwhelming. The hospital hallways, lined with patients and their families at first felt claustrophobic.

But, day by day, we began to adopt a more quotidian attitude and a studious enthusiasm for learning. The hallways began to feel less crowded and the patient’s problems less emotionally overwhelming as our focus shifted from the environment to individual signs, symptoms and treatments.

Foucault would have referred to this as the development of the “medical gaze.” For us, though, it was not a philosophical construct but a pragmatic response to one of the most real and tearing realities of medical education: that learning depends not only on the willingness of patients to share their personal problems with us but also on the student’s ability to occasionally look beyond the personal circumstances of the person suffering to understand the scientific principles that underlie the problem. Medical students enter their training full of empathy for the individual patient. Somewhere along the way they begin to realize that this is not enough, and that the real challenge lies in finding a good mix of empathy and objectivity.

We stepped into the treatment room like four scared, shocked kittens comically dolled up in white coats. The consultant strode in and without pause began to take a history from the patient and his family, trying to piece together the story. None of us foreigners spoke Lugandan, and so we had no choice but to wait until the consultant could translate for us. As we watched meekly, the patient, in evident discomfort, lay supine, knees bent, head thrown back, with the freshly healed stigmata of a dog's bite on his distal forearm.

Soon satisfied with the history, the consultant gave us a quick summary. The man had received the bite while defending his daughter from the attack of a wild dog. Thinking nothing of the minor wound on his arm, and daunted by the prospect and the cost of coming to Mulago hospital, he had dismissed the injury until he began to develop his current symptoms: extreme terror, laryngeal spasm and hydrophobia.

A classic case.

## Rabies

A uniformly fatal infection, still common in many parts of the tropics, that is caused by the rabies virus ... . [O]nce clinical symptoms have appeared, the patient will die. However, if the infection is caught soon after transmission and before the onset of clinical symptoms, rabies can be prevented by post-exposure vaccination.

There is currently no effective treatment for a person who is showing signs and symptoms of rabies infection. In this situation, management is symptomatic with sufficient sedation and analgesia to relieve pain and terror.

Vaccination within days of exposure is 100% effective in preventing the progression of the infection to encephalitis. However, the cheap Semple vaccine that is used most widely in the developing world is itself capable of initiating encephalitis ... . [T]he vaccine can produce severe CNS disease with a 3% mortality.

Oxford Handbook of Tropical Medicine, second edition, pp. 410–13

The medical plan for the bitten man was continued observation, accompanied by any palliation the hospital resources could afford. We never found out whether he was told of his prognosis, or whether any effort would be made to find his daughter and initiate the post-exposure rabies vaccine protocol before it was too late. Such follow-up was not always possible in the chaos of the hospital.

The consultant volunteered to give us a lift so we wouldn’t have to walk home in the dark. For four white bzungu it was probably a risk not worth taking. Not even the jostling of his car over the deep potholes of Wandegeya road could shake the image of the bitten man or his story from my head.

Places like Kampala are, for me at least, the places where all of the most enraging injustices of the world are presented, bare, raw, and worn to the bone. Seeing people succumb to diseases and injuries that in high-income countries would be dealt with quickly as minor inconveniences turns each death and disability into a tragedy all the more powerful because it is so obviously preventable. Although such inequities may be the visible vestiges of huge economic and political systems bearing their full weight on the oppressed, to see such inequity first-hand fills one with a sense of personal grief and urgency to act.

There is also often a sense of guilt. Walking through the hospital, armed with gloves, gowns and masks, ingesting my malaria prophylaxis daily while the local doctors and patients struggled to scrape together the few dollars it takes to get a needed x-ray, I was acutely aware of my advantaged place in those systems. Part of me wanted not to accept anything more than what the people there, who had taken us into their homes, fed us, and taught us, could possibly get themselves. Another part of me knew that, like it or not, my position in the world was somehow different from that of the others in the hospital.

## Home

I woke from my sleep that Saturday evening in sheets soaked with sweat, as Sarah and I listened to the shrill squeals of bats overhead.

We were in a small cabin at Murchison Falls National Park in the northwest of the country, having completed a two-day safari in and about the Nile River. After two weeks spent mostly in the cramped quarters of Mulago Hospital and the smoggy, congested streets of Kampala, a leisurely, planned tour of the park to see some wildlife was a welcome diversion.

All of that came to a sudden end with the flap of a pair of leathery wings in the air around our beds. Having just two nights earlier seen a man in the early throes of rabies, neither Sarah nor I were thrilled at the possibility of meeting that same fate ourselves. We spent a frantic night in the pitch-dark room, terrified that maybe we had been bitten. Bat bites, I had learned, were often invisible and painless, revealing themselves only once it was too late.

In some ways, the role of the physician in health and disease is simple.

The explicit teaching is this: Patients arrive with a chief complaint, which sometimes clearly identifies the disease but is sometimes misleading. It is the job of the physician to elicit the important symptoms and signs and evaluate their importance to determine the most likely diagnoses and the patient’s likely prognosis. This information should be clearly and empathetically communicated to the patient. Then, together, the patient and the doctor have to evaluate the risks and benefits of various treatments, selecting one that will meet the patient’s needs and goals.

The implicit teaching is this: As physicians, we are equipped with knowledge and skills that many patients do not have, and by which we may be of assistance. Although the patient’s perspective is vital in determining the goals of treatment, medical professionals should retain a sense of objectivity to more accurately determine diagnoses and the relative merits of various treatments.

In short, there is an “us” and a “them.” The “other” persists.

Of course, all notions of objective risk assessment tend to go out the window when physicians try to apply them to themselves. Medical student syndrome — “hypochondriasis of medical students” — is a particularly acute example of this. It is not uncommon for students to diagnose themselves with an aortic dissection, gallstones, peptic ulcer disease, or any one of a number of metabolic or endocrine derangements, depending on which disease they happen to be studying at the time. When it comes to themselves, physicians seem unable to stick to the objectivity and rules they so coolly apply to others.

So it may be, too, with our ideals of health and justice.

The ride home the next morning took us on sunny winding roads through the Rwenzori mountains and their lush fields of tea and tobacco. Sarah was quiet for the entire ride, partly because of the Gravol, but certainly also because of her worry about what might happen after the 9- to 20-day rabies incubation period we had so recently learned about, and the risks of the local Ugandan rabies post-exposure protocols.

When we arrived back at our hostel in Kampala that night, I called my mother back home in Hamilton and asked for her advice. She made a few calls to travel doctors in the area and quickly found out that there was another local option, a private clinic called “The Surgery,” the longest continually held practice in Kampala. Started in the 1940s by ex-pat British GPs while Uganda was still under colonial rule, the clinic has been used by international travellers to Kampala for whom nothing less than the “Western standard” will suffice.

After speaking to a nurse at the clinic, we soon found that although The Surgery did not use the Semple post-exposure vaccine, the protocol they followed was substantially different from the one we used in Canada — in particular, because it did not include the immunoglobulin injection during the initial treatment phase. The nurse told us that, despite this fact, they’d never had any reports of the protocol’s failure in all their many years of practice.

If it is a tragedy each time a Ugandan dies from a preventable, treatable illness, then what is it called when a visiting medical student witnesses that tragedy and then promptly visits a clinic that costs ten times as much for a much more minor condition? Reasonable precaution? Irony?

To me, it felt like hypocrisy. Most Canadians point to the ideals of medicare as one of Canada’s greatest civil achievements: a system of care based on need rather than ability to pay. As a society, we pool risk, share benefits, and sacrifice autonomy for the sake of others in society, usually complete strangers. What makes us do so for the sake of strangers within our country but not elsewhere in the world?

Economics? Geography? Futility?

“You need that immunoglobulin. All of the doctors here at home agree.”

Despite my protests, consults with the local Mulago doctors, and personal research, I knew that the doctors at home were probably right. If I had been bitten by the bat, and if it had carried rabies, the only rational thing to do would be to minimize the chances that the disease would spread throughout my system. The severe consequences of infection were too great to avoid taking all necessary precautions.

Unfortunately, even if I managed to procure local immunoglobulin, there were still complications. The concentration of IG in human blood is so low that to create the substantial dose necessary for a rabies protocol requires extracts of blood from multiple donors. In Uganda, with rates of HIV infection in urban areas reported to be as high as 30%, receiving such a concentrate of blood products was a risky prospect in itself.

“Look, health care is practised differently in Uganda than it is in Canada ... and since you can’t get the im- munoglobulin there, your best bet really is to come home. So quit stalling in Kampala and start making arrangements to fly back as soon as possible.”

I hung up the phone, paused, and started to formulate my plans.

Many of the doctors we worked alongside at Mulago had actually spent a portion of their training in Hamilton, Ontario. There was a sense of camaraderie and shared experience between us as we traded fish-out-of water stories. It was not uncommon for the doctors to discuss a patient’s assessment and plan with finality, and then direct additional comments toward us: “Of course, back in Canada you would likely deal with the problem using ——— since you’d have the resources for it. Here we can’t do that so we have to make do.”

Watching the skilled hands and minds of the doctors as they worked on the crowded wards was a revealing experience. I can’t really imagine what it was like for them, having to come up with ad hoc, “good-enough” solutions for complicated problems after having studied and practised in Canada, where we investigate every symptom to the nth degree with extensive follow-up. Were they driven crazy knowing all too well what they were missing? Did they think it unfair that, in Canada, medical students wielded resources that even the top doctors in Mulago would envy?

None of the doctors or nurses or other professionals I met working at the hospital, or even any of the patients, indicated that sentiment even once. Rather, there seemed to be a resignation toward our unequal relationships: “Even if we don't have the resources you do at home, come, learn from us and let us learn from you, too; we understand why you look after yourselves with such caution.”

The inequities that separate us may be substantial, but they do not have to be barriers to meaningful relationships.

A few days later, as my plane glided off the runway at Entebbe International Airport, my thoughts swarmed with all of the people, places and events I had witnessed during the past few weeks.

After two airplane meals I started to think about how I was going to organize the 28 days of rabies vaccinations around my remaining clinical electives.

By the time I arrived home 23 hours later, I had comfortably slipped back into my daily routines. I almost felt as though nothing had changed, that maybe I had never left home at all.

But Mulago is still there. The doctors are there. And the bitten man might still be there.

I’ll never know.

